# Novel Orthohantavirus Associated with Hantavirus Pulmonary Syndrome in Northern Argentina

**DOI:** 10.3390/v17050717

**Published:** 2025-05-16

**Authors:** Carla M. Bellomo, Sebastian Kehl, Daniel Oscar Alonso, Walter López, Flavia Cassinelli, Rocío María Coelho, Gabriela Bravo, Sara Aguirre, Marcela Dib, Natalia Periolo, Concepción Toscano, José Gil, Francisco García Campos, Ignacio Ferro, Valeria Paula Martinez

**Affiliations:** 1 Instituto Nacional de Enfermedades Infecciosas (INEI)—Administración Nacional de Laboratorios e Institutos de Salud (ANLIS) Malbran, Buenos Aires C1282, Argentina; skehl@anlis.gob.ar (S.K.); daniel.alonso.89@gmail.com (D.O.A.); rcoelho@anlis.gob.ar (R.M.C.); nperiolo@anlis.gob.ar (N.P.); pmartinez@anlis.gob.ar (V.P.M.); 2 Instituto de Investigaciones de Enfermedades Tropicales-IIET, Universidad Nacional de Salta (UNSa), Salta A4400, Argentina; wlopezbio@gmail.com; 3 Instituto de Ecorregiones Andinas (INECOA), Consejo Nacional de Investigaciones Científicas y Técnicas (CONICET), Universidad Nacional de Jujuy (UNJu), San Salvador de Jujuy Y4600, Argentina; flacassinelli@gmail.com (F.C.); ignacioferro@gmail.com (I.F.); 4 Hospital Señor Del Milagro, Salta A4400, Argentina; arbovirus.hantasalta@gmail.com (G.B.); marcedib@hotmail.com (M.D.); cunchitoscano@gmail.com (C.T.); 5 Instituto de Investigaciones en Energía No Convencional (INENCO), Consejo Nacional de Investigaciones Científicas y Técnicas (CONICET), Universidad Nacional de Salta (UNSa), Salta A4400, Argentina; saagguirre@gmail.com (S.A.); jgil.conicet@gmail.com (J.G.); 6 Dirección General de Coordinación Epidemiológica, Ministerio de Salud Pública de Salta, Salta A4400, Argentina; pacogarciacampos@yahoo.com.ar

**Keywords:** orthohantavirus, hantavirus pulmonary syndrome, Salta, Argentina, zoonosis, infectious diseases, Guachipas, rodents

## Abstract

In this work, we performed the genetic characterization of a new variant of orthohantavirus associated with a fatal case of hantavirus pulmonary syndrome, outside the known endemic region, in northwestern Argentina. We first confirmed an orthohantavirus infection by ELISA, testing for the detection of IgM and IgG antibodies. Then, we extracted RNA from 100 microliters of serum, the only sample available, followed by RT-PCR. The amplicons were sequenced using Sanger and next-generation sequencing technology. We obtained partial sequences of 1253 bp, 799 bp and 1675 bp from the S-, M- and L-segments, respectively, showing low sequence identities with all the previously characterized hantaviruses (10.9%, 13.5% and 15.1% of the divergence, respectively). The phylogenetic analysis showed that this virus belongs to the *Orthohantavirus andesense* species (ANDV), and among the ANDV-like variants, it is more closely related to the Lechiguanas clade. Similar percentages of divergence were considered sufficient to distinguish AND-like variants in previous works. As the patient had no travel history before the onset of disease was reported, we conducted rodent surveys to confirm the presence of reservoirs. The rodent assemblage was compatible with the transitional zone among different ecoregions (Yungas, Chaco and Monte). Moreover, one of the species captured, *Oligoryzomys flavescens*, was previously described as a reservoir of hantavirus. This species may either host several variants across its range or encompass a species complex, as proposed by some authors.

## 1. Introduction

Hantavirus pulmonary syndrome (HPS) is a zoonotic disease caused by viruses classified under the genus *Orthohantavirus*, subfamily *Mammantavirinae*, family *Hantaviridae*, and order *Elliovirales*. They are enveloped viruses with tri-segmented single-stranded RNA genomes consisting of small (S), medium (M) and large (L) segments and encoding the structural protein nucleoprotein N, the glycoproteins Gn and Gc, and the viral polymerase [1,2,3]. Several species of sylvan rodents act as reservoir hosts in nature. Humans become infected through the inhalation of aerosolized viral particles generated by infected rodents. Andes virus (ANDV) was the first etiologic HPS agent identified in Argentina [4]. ANDV and several similar variants, here referred to as AND-like, are classified under the species *Orthohantavirus andesense* and have different geographic distribution patterns in Argentina [5]. Orán (ORNV) is the most prevalent in the northwestern endemic region, while Bermejo (BERV) and Buenos Aires (BAV) are infrequent [6,7,8,9]. Laguna Negra virus (*Orthohantavirus mamorense*) is also rarely found in human cases and rodents in the region [10].

**Figure 1:**
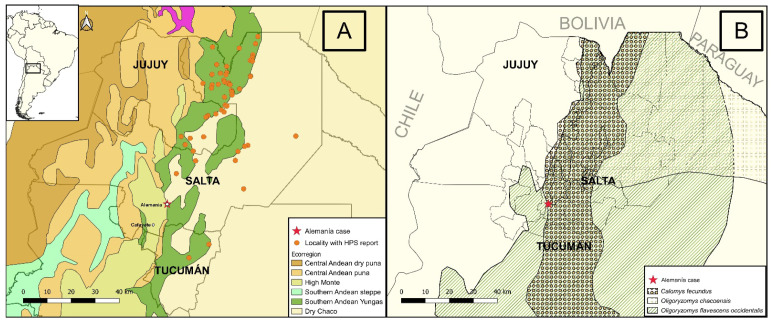
Map of northwestern Argentina. (**A**) Locations where hantavirus pulmonary syndrome (HPS) was reported in northwestern Argentina between 1997 and 2023; circles indicate the locality of the HPS reports, and the red star the case reported in Alemanía. The biogeographic regions are represented by colors and were obtained from Olson 2001. (**B**) Distribution of rodent species previously associated with hantavirus; data were obtained from the Argentine Society for the Study of Mammals. Maps were created with the QGIS 3.16 geographic information system. Open Source Geospatial Foundation Project (http://qgis.osgeo.org).

The HPS endemic region of northwestern Argentina (NWA) accounts for almost 50% of HPS cases in the country, while the rest are unequally distributed among the other three regions [8,9]. Most cases occur in the Yungas rainforest ecoregion, in the foothills and lowlands of the northern Salta and eastern Jujuy provinces, while only a few cases are reported in the semiarid woodlands of Chaco ecoregion in northeastern Salta province [11,12] (Figure 1). Genetic characterization of viruses from rodents in the endemic region of NWA led to their association with reservoir host species: ORNV with *Oligoryzomys chacoensis*, BERV with *O. flavescens occidentalis*, and LNV with *Calomys fecundus* [8,9,10].

HPS is characterized by a long incubation period (9 to 45 days after exposure), a short prodrome and rapid progression to respiratory failure and often death [11,13]. Unfortunately, there is no specific treatment or vaccine approved for HPS.

In this study, we report the identification and genetic characterization of a novel AND-like variant associated with a fatal case of HPS outside the known endemic region of NWA. Additionally, we conduct rodent surveys to confirm the presence of reservoirs. The identification of rodent reservoirs is of key importance for the establishment of accurate preventive measures and for the understanding of the ecoepidemiology of hantavirus infections.

## 2. Materials and Methods

### 2.1. Case Definition

A suspected case of HPS is defined as a patient who resides in or reports a recent travel history to an endemic region with persistent fever (>48 h), headache, myalgia, and/or gastrointestinal manifestations (e.g., abdominal pain, vomiting, and/or diarrhea), with the addition of any advanced respiratory manifestations. A confirmed case is defined as a person with ELISA-specific IgM and IgG antibodies in a serum sample [5,12]. The ELISA test is based on viral nucleoprotein and has been found to be capable of detecting all the reported hantavirus variants in South America. The test has been shown to have a diagnostic sensitivity and specificity of 96.6% and 90.6%, respectively [12].

An outbreak of hantavirus is defined as follows: (1) the emergence of an autochthonous case in an area where no cases have been previously reported; (2) a large increase in cases in an endemic region; or (3) the identification of grouped cases (National Ministry of Health) [14].

### 2.2. Viral Characterization

Viral RNA detection and genetic characterization were conducted through the extraction of the total RNA from a serum sample (for the HPS case) or a lung sample (for rodent tissues) using a Zymo Direct-Zol kit followed by reverse transcription–polymerase chain reaction (RT-PCR) using a SuperScript III RT-PCR kit. The amplicons were sequenced using Sanger or next-generation sequencing (NGS) technology for rodent or human samples, respectively; both assays were performed as previously described [15].

For the phylogenetic analysis, the nucleotide sequences were first aligned using ClustalW, and maximum likelihood phylogenetic trees were constructed using IQ-Tree v2.0, using the best-fit nucleotide substitution models determined by Model Finder [16,17]. An ultrafast bootstrap of 1000 replicates was used in the tree construction to assess the node support. Pairwise nucleotide comparisons were performed with published sequences and/or sequences available at the National Reference Laboratory for Hantavirus (NRLH).

### 2.3. Epidemiological Investigation and Environmental Survey

The study area was defined as the residence of the HPS case and nearby places identified as risk sites for rodent exposure in Alemanía, Department of Guachipas, Salta (Figure 1). The Guachipas Department supports diverse habitats with elevations ranging from 995 m to 3176 m above sea level, an annual average precipitation of 692 mm, and a mean annual temperature of 17.5 °C, spanning from 5.3 °C (minimum temperature of the coldest month) to 25.8 °C (maximum temperature of the warmest month) [18].

### 2.4. Georeferencing

Cumulative HPS case data were obtained from records compiled by the NRLH, together with the distribution of reservoir hosts obtained from the Argentine Society for the Study of Mammals [19,20,21], and the terrestrial ecoregions obtained from Olson et al. 2001 were spatially represented using the software QGIS 3.16 (SRC: EPSG 4326-WGS 84) [22]. The political division of Argentina was obtained from the National Geographic Institute of the Argentina Republic (https://www.ign.gob.ar, (accessed on 20 August 2024)). The vegetation units of the native forest were taken from the geospatial information platform of the spatial data infrastructure of the Province of Salta (http://geoportal.idesa.gob.ar/, (accessed on 20 August 2024)). The Normalized Difference Vegetation Index (NDVI), including the years 2000 to 2012, was obtained from R package MODIStsp. The digital elevation model for Argentina was downloaded from the DIVA-GIS repository.

### 2.5. Rodent Trapping and Characterization

A reservoir trapping study was conducted in September 2024, using 318 Sherman live traps for small rodents, with 1086 trap nights over four nights. The survey methods followed the animal care procedures outlined by the American Society of Mammalogists [23]. All the animals were handled according to the Argentine law for the protection of animal welfare (Penal Code, law Nº 14.346), following safety standards based on the “Biosafety Guidelines for Working with Small Mammals in a Field Environment” [24]. Blood samples were collected by cardiac puncture and tissue samples by systemic necropsy. The morphometric and reproductive features, and the presumptive species, were recorded for each captured animal.

The presence of IgG-specific antibodies in rodent blood samples was determined by ELISA as described previously [5]. To verify the morphological host species determination and to assess the potential geographic variation of *Oligoryzomys* species, a portion of the mitochondrial cytochrome b (cytb, 1140 bp) was sequenced. The total DNA was extracted from a liver sample and the National Center for Biotechnology Information (NCBI) Basic Local Alignment Search Tool (BLAST) was used for species identification.

## 3. Results

### 3.1. HPS Case Confirmation

On 13 July 2024, a 16-year-old woman was admitted to Papa Francisco Hospital, in Salta province, with fever, cough, tachypnea, hepatomegaly and oliguria. The patient complained of fever, headache and abdominal pain since the 9th of July. After a rapid progression to respiratory failure and shock, the patient died (less than 24 h after her admission). The clinical laboratory findings were compatible with hantavirus infection, revealing hemoconcentration (53%), leukocytosis (13,900 cells/mL), neutrophilia (90%) and thrombocytopenia (52,000 cells/mL). The initial serological screening for hantavirus infection was subsequently performed at El Milagro Hospital (Salta), a local node of the Hantavirus National Laboratory Network, where low titers of IgM and IgG for ANDV were detected. A serum sample was sent to the NRLH, Instituto Nacional de Enfermedades Infecciosas ANLIS “Dr. Carlos G. Malbrán”, where hantavirus infection was confirmed by ELISA with IgM capture and IgG.

### 3.2. Genetic Characterization

Despite the fact that EDTA-containing blood is the recommended sample for molecular diagnosis and HPS investigation, the total RNA was extracted from a 100-microliter serum sample, the only one available, due to the suspicion of hantavirus infection that arose following the death of the patient. The amplicons obtained by RT-PCR were subjected to next-generation sequencing, obtaining partial sequences of 1253 bp, 799 bp and 1675 bp from the S-, M- and L-segments, respectively. Pairwise nucleotide comparisons showed low sequence identities with all the previously characterized hantaviruses. Comparisons of the full length of S- and M-segments’ partial fragments revealed the highest identities with ARG-OF.53 (*Orthohantavirus andesense*, GenBank accession number OR890439), a sequence obtained from an *O. flavescens* captured in the Humid Chaco ecoregion about 950 km eastwards of the present case. For the L-segment, the highest identity found was 84.9% with an LECV recovered from an HPS case reported in the province of Entre Ríos about 1400 km eastwards of the present case (ER19-SP1693, Acc. No. PP151172). Higher identities were found with very short published S-segment sequences (468 bp) of *Oligoryzomys* spp. captured near the area of the preset case residence (Table 1).

**Figure 2:**
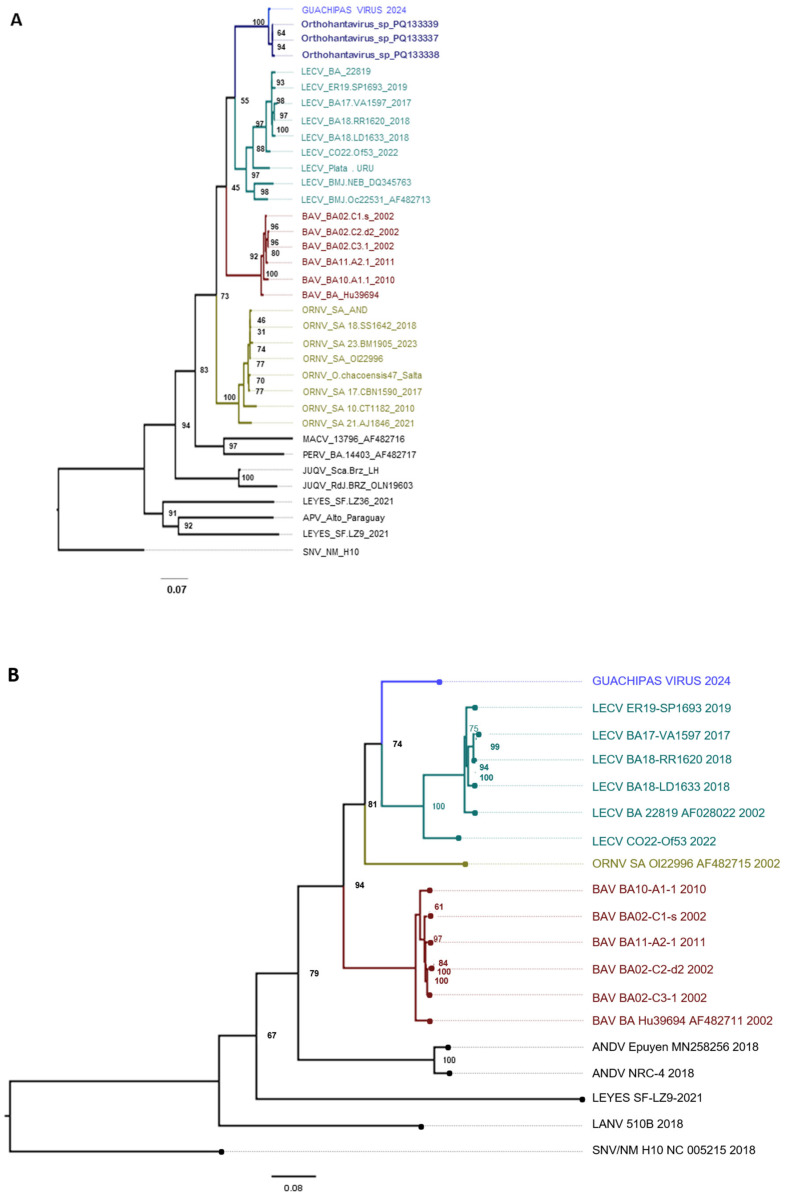
Phylogenetic tree of the partial S-segment (**A** panel) and M-segment (**B** panel). Best-fit model according to the BIC: TIM2 + F + I + G4. The phylogenetic relationships of representative Argentinian viruses in the *Hantaviridae* family were reconstructed using the maximum likelihood method. The bootstrap values defined for a run of 1000 repetitions are displayed on each node. Clades with different colors indicate different species. The newly sequenced taxon is highlighted in blue. LECV: Lechiguanas virus; ORNV: Orán virus; BAV: Buenos Aires virus; ANDV: Andes virus; MACV: Maciel virus; PERV: Pergamino virus; JUQV: Juquitiba virus; APV: Alto Paraguay virus; SNV: Sin Nombre virus; LANV: Laguna Negra virus.

The phylogenetic reconstruction for the S- and M-segments revealed that the new virus grouped with the AND-like viruses but clearly constituted a separate lineage, segregated into a different branch of the monophyletic LECV clade, well supported by the bootstrap values (Figure 2).

### 3.3. Eco-Epidemiological Features

The case reported in Alemanía, Department of Guachipas, is the first hint of the circulation of hantavirus in the area. Alemanía is located in a transitional zone between the ecoregions of the High Monte desert, Yungas Forest and Dry Chaco, but the dominant vegetation has clear affinities to the Chaco ecoregion. The area is crossed longitudinally by the river “Las Conchas”. The epidemiological investigation revealed that the infection probably occurred while cleaning an abandoned cabin surrounded by native forest, near the family house in the locality of Alemanía, a very small town of less than 100 inhabitants built around an abandoned railway station, immersed in an environment of native vegetation (Figure 1). But note that the family also frequented Cafayate, a touristic village located 80 km southwards, known for its beautiful landscapes and wine tours. The town houses extend 1 km southwards, scattered in a narrow ravine (“Quebrada de las Conchas”) on the riverside and surrounded by rocky mountains with dense xerophytic forest cover. Most of the people who live in the village are artisans who sell their products on the side of National Route 68 to tourists driving between Cafayate and Salta city. The topography becomes flatter to the north of Alemanía, where agriculture becomes the principal economic activity, mainly horticulture, pastures, cattle and tobacco interspersed with the native forest of the Chaco, as described in areas where HPS is endemic.

### 3.4. Rodent Community Description

As a result of the survey, 22 rodents were collected, with a trapping success rate of 2.1%. The species identified were *O. flavescens occidentalis* (4.55%), *O. brendae* (22.7%), *Graomys griceoflavus* (54.5%), *G. domorum* (14%) and *Calomys musculinus* (4.55%). Although 27.25% of the captured rodent species (*Oligoryzomys* spp.) had previously been reported as reservoirs for hantavirus, no seropositive specimens could be recovered. For the rodent belonging to a genus previously described as a hantavirus reservoir, an RT-PCR (s-segment coding region) was conducted, yielding negative results.

## 4. Discussion

A previous study postulated risk areas for orthohantavirus transmission beyond the currently known HPS endemic region and suggested that the disease is underreported [25]. Although the occurrence of the case presented in this study is consistent with this hypothesis, it is also possible that infections are infrequent in this area. While orthohantavirus transmission between rodents occurs predominantly under the shelter of their nests or during fights, the transmission to humans could be limited by high solar radiation and the low vegetation cover [26,27,28]. The area with the highest incidence of HPS in Argentina, Oran Department, has an average Normalized Difference Vegetation Index (NDVI, an indicator of vegetation photosynthetic activity) of 0.64 and an elevation of 623 m above sea level. In contrast, Guachipas Department has an average NDVI value of 0.53 and an elevation of 1789 m above sea level. The lower vegetation cover and more intense radiation due to the higher altitude can lead to faster inactivation of the virus in the environment. It is worth noting that the most probable infection place was an abandoned house while cleaning. The virus could have prolonged its viability under this condition. Furthermore, a recent work presented an HPS risk transmission map for NWA based on ecological niche models of the reservoir species together with climatic and environmental variables, which predicted the presence of three orthohantavirus reservoir species in the area: *O. f. occidentalis*, *O. chacoensis* and *C. fecundus* [25]. The former species was captured during the rodent survey. Although the sampling effort was low to fully characterize the rodent community, the presence of different *Oligoryzomys* species in syntropy could indicate the co-circulation of heterologous hantaviruses.

In the present study, a new pathogenic orthohantavirus was characterized from a serum sample of a patient who died after fulminant disease. The overall genetic divergence from all the previously identified hantaviruses was greater than 10%. Similar percentages of divergence were considered sufficient to distinguish AND-like variants in previous works [5,15,29,30]; therefore, we propose to name the new strain Guachipas virus. The phylogenetic analysis showed that Guachipas virus belongs to the *Orthohantavirus andesense* species, and among the AND-like variants, it is more closely related to the LECV clade. The present analysis was based on the most conserved region of the viral segments. It is therefore postulated that an analysis of the whole genome would reveal greater distances. A short sequence from the S-segment of an uncharacterized viral strain recovered from *Oligoryzomys* spp. specimens, not determined at the specific level, that were caught in 2014 at a nearby site, 68 km north of Alemanía, clustered together with Guachipas virus, suggesting a high degree of relatedness; however, the limited genomic information does not allow further comparisons. Nevertheless, the available evidence suggests that the reservoir of Guachipas virus is likely one of the *Oligoryzomys* species captured and identified in the present study.

Human habitat disturbance is predicted to increase the hantavirus prevalence within an ecosystem. Disturbed habitats tend to favor more generalist species that tolerate and adapt to ecological changes [26]. Because *Oligoryzomys* species are generalists, they are prone to interact with humans in rural settings. In this study, *O. flavescens occidentalis* and *O. brendae* were caught exclusively in the alfalfa crop and in the chicken coop adjacent to the house of the HPS case. These economic activities would indirectly provide resources for hantavirus reservoir species. The nearest endemic region displays a comparable pattern of variation in rodent abundance over time. There is a positive relationship between HPS cases and rodent abundance, with a three-month delay, with a rapid increase and decrease of rodent abundance [28]. Given the HPS incubation period (9–45 days) and the fact that the rodent survey was conducted two months after the case was reported, the relatively low rodent abundance and the subsequent absence of positive individuals are not surprising.

The description of this new pathogenic variant highlights the great diversity of hantavirus present in NWA. There are currently five orthohantavirus circulating in NWA: LNV, ORNV, BERV, BAV and Guachipas virus. This is relevant when considering that spillover has allowed the emergence of new species associated with hantavirus transmission [31]. As hantaviruses have segmented genomes, the co-circulation of more than one variant allows the reassortment of their genomes, which occasionally could give rise to progeny with increased fitness. Natural reassortment events were reported for Sin Nombre, Puumala, Dobrava-Belgrade, Hantaan and Imjin virus, while in vitro reassortment was reported [32,33,34] Whilst typically deleterious, documented cases exist of reassortment in other viral families, resulting in altered virulence and facilitated emergence of novel hosts [30,35].

In summary, this work describes the circulation of a new pathogenic orthohantavirus in a transitional area of NWA, where the High Monte desert, Yungas Forest and Dry Chaco ecoregions converge. Further studies, including rodent captures in Cafayate, are needed to identify the reservoir species and to estimate the incidence of hantaviruses in the human population. The new evidence presented here will enable appropriate interventions to promote clinical suspicion, improve detection and thereby prevent the emergence of hantavirus in the area.

## Figures and Tables

**Table 1:** Percentage of pairwise nucleotide and amino acid divergence between the amplified viral sequences from the patient residing in Alemanía, Department of Guachipas, and the most similar hantaviruses from GenBank.

	S-Segment (1253 bp) Divergence				M-Segment (824 bp) Divergence				L-Segment (1735 bp) Divergence				Virus/Strain
	Nucleotides		Aminoacid		Nucleotides		Aminoacid		Nucleotides		Aminoacid		
	Acc. No.	(%)	Acc. No.	(%)	Acc. No.	(%)	Acc. No.	(%)	Acc. No.	(%)	Acc. No.	(%)	
ARG-OF.53	**OR890439**	**10.9**	WZP32982	1.5	**OR890440**	**13.4**	WZP32983	4.8	UP	-	UP	-	Lechiguanas
ER19-SP1693	OR908896	11.5	WPW61495	1.3	OR987856	14.7	XGD07576	5.8	XGD07567	15.1	XGD07565	1.1	Lechiguanas
Oc22531	AF482713	11.8	AAL82648	1.2	AF028025	NA	AAB87911	NA	UP	-	UP	-	Lechiguanas -Bermejo
Arg-h484	OP555730	12.8	WNV26842	1	OP555731	16.2	WNV26843	6.8	OP555736	17.7	WNV26848	2	Buenos Aires
Ol22996	AF482715	13.3	AAL82650	2	AF028024	17.5	AAB87910	10	UP	-	UP	-	Orán
FBV554	JF750419	15	AEO51745	2.7	JF750422	NA	AEO51748	NA	UP	-	UP	-	Tunari
TK184992	OR184959	16.8	WNR62054	5	OR184986	21.5	WNR62081	11.5	OR184993	19.9	WNR62163	3.9	Juquitiva
ARG-Epuyen	MN258239	16.5	QNQ18256	1.2	MN258205	20.6	QNQ18222	11.2	MN258172	19	QNQ18189	2.7	Andes

NA: The only published sequences are partial; the published fragment belongs to a different nucleotide range than the one amplified in this work. UP: unpublished. Acc. No.: GeneBank accession number. Names in bold in the table indicate the sequence with higher similarity to the Guachipas sequences. The GenBank accession numbers of the Guachipas virus are: PQ181488, Q356349 and PQ373861 for the S-, M- and L-segment, respectively.

## Data Availability

The original data presented in the study are openly available in the NIH genetic sequence database (GenBank). Raw data were generated by the INEI-ANLIS “Dr. C. G. Malbrán”. Derived data supporting the findings of this study are available from the corresponding author, C.B., upon request.

## Abbreviations

HPS	Hantavirus pulmonary syndrome
NRLH	National Reference Laboratory for Hantavirus
RNA	Ribonucleic acid
DNA	Desoxyribonucleic acid
RT-PCR	Reverse transcription–polymerase chain reaction
ELISA	Enzyme-linked immunosorbent assay
ANDV	Andes virus
BERV	Bermejo virus
BAV	Buenos Aires virus
LECV	Lechighuanas virus
ORNV	Oran virus
LNV	Laguna Negra virus
NWA	Northwest Argentina
NGS	Next-generation sequencing
NCBI	National Center for Biotechnology Information
BLAST	Basic Local Alignment Search Tool
